# To what extent are anorectal function tests comparable? A study comparing digital rectal examination, anal electromyography, 3-dimensional high-resolution anal manometry, and transperineal ultrasound

**DOI:** 10.1007/s00384-022-04304-6

**Published:** 2023-01-16

**Authors:** L. Dekker, D. A. van Reijn-Baggen, I. J. M. Han-Geurts, R. J. F. Felt-Bersma

**Affiliations:** 1 Department of Surgery, Proctos Clinic, Bilthoven, The Netherlands; 2 Department of Surgery, Amsterdam University Medical Centers, Location AMC, Amsterdam, The Netherlands; 3 Department of Urology, Leiden University Center, Leiden, The Netherlands; 4 Department of Gastroenterology & Hepatology, Amsterdam University Medical Centers, Location VU, Amsterdam, The Netherlands

**Keywords:** Digital rectal examination, Anal manometry, 3D-HRAM, Balloon expulsion, Transperineal ultrasound, Electromyography, Pelvic floor physical therapy, Biofeedback

## Abstract

**Background:**

Anorectal function tests are helpful for objective investigation of anorectal (dys)function. A variety of tests are available, but there is no recommendation when to perform which test. Furthermore, which test is the most accurate is controversial and the correlation between these tests is not very clear. The aim of our study was to examine the correlation of anal pressures and the possibility to diagnose pelvic floor dyssynergia between digital rectal examination (DRE) and several anorectal function tests.

**Methods:**

Between January 2020 and April 2022, all men and women aged 18 to 80 years, treated at the Proctos Clinic, who were referred for pelvic floor physical therapy (PFPT) by the surgeon and underwent anorectal function tests, were included. DRE was performed to establish the anal pressure at rest and during squeeze and straining. Anorectal function tests included 3D high-resolution anal manometry (3D-HRAM), balloon expulsion test (BET), transperineal ultrasound (TPUS), and surface electromyography (s-EMG).

**Results:**

A total of 50 patients, 37 (74%) females, were included. Median age was 51 years. Twenty-three (62%) females had a history of two or more vaginal deliveries. The most frequent reason for referral for PFPT was fecal incontinence in 27 (54%) patients. The assessed pressures and pelvic floor function measured with DRE by the surgeon and the pelvic floor physical therapist during rest, squeeze, and straining correlated in 78%, 78%, and 84%, respectively. Correlation between DRE and 3D-HRAM or s-EMG was better for squeeze pressures than resting pressures. The correlation between s-EMG and 3D-HRAM was better during squeeze than at rest with an agreement of 59% and 37%, respectively.

**Conclusion:**

DRE by an experienced investigator is of sufficient value for daily clinical practice to detect dyssynergia and to measure sphincter tone. Commonly performed anorectal function tests correlate poorly with DRE and with other anorectal function tests. When conservative treatment fails, further investigation is warranted; however, these results should be interpreted with caution.

**What does this paper add to the literature?:**

Anorectal function tests such as the 3D high-resolution anorectal manometry, balloon expulsion test, surface electromyography, and transperineal ultrasound are all frequently performed in the diagnostic workup in patients with defecation disorders. No previous study has compared these tests regarding their outcomes, nor has the interrater agreement been measured regarding the digital rectal examination by two experienced observers. Furthermore, transperineal ultrasound is in all probability not frequently used and therefore underexposed in the diagnostic workup of patients with dyssynergic defecation.

## Introduction

Anorectal function disorders like fecal incontinence and chronic constipation are very common. Generally, a conservative approach with lifestyle advices, fibers, laxative, and pelvic floor physical therapy will improve complaints in many patients. When unsuccessful, or the underlying cause seems unclear, these patients are referred to a specialist for further evaluation of anorectal function and possible therapy [1]. Besides digital rectal examination (DRE), a variety of tests are available to evaluate anorectal function. One may then objectively assess, e.g., low or high tone of the anal sphincter, paradoxical contraction, or inadequate relaxation of the pelvic floor.

Available tests are, for example, anorectal manometry (ARM), 3-dimensional high-resolution anorectal manometry (3D-HRAM), balloon expulsion test (BET), surface electromyography with or without an intra-anal probe (s-EMG), transperineal ultrasound defecography, and the classical defecography. Although some studies suggest that DRE alone is a useful tool to identify anorectal disorders [2, 3], others propose that anorectal function tests objectively evaluate anorectal function and might provide a predictive value for treatment results and influence management [4–9]. Which anorectal function test is the most accurate is under debate.

The s-EMG with intra-vaginal or intra-anal electrode probes is commonly utilized by the pelvic floor physical therapist to confirm DRE and evaluate therapy [5, 10]. ARM is often considered the gold standard to measure anal pressures; however, lack of reproducibility mentioned in several studies makes the test questionable [11–16]. Few studies compared ARM with anal s-EMG and showed limited concordance [17–19]. A more recent study compared ARM with DRE to determine dyssynergia and concluded that there was a moderate agreement [20].

According to the ROME IV criteria, dyssynergia is established by two out of three anorectal function tests: first, abnormal anorectal evacuation pattern measured with ARM or s-EMG; second, abnormal BET; and third, impaired rectal evacuation diagnosed on imaging studies (e.g., defecography) [7]. Furthermore, examinations as DRE and transperineal ultrasound are not mentioned in this context and a clear gold standard for one of these tests is not suggested. One could wonder whether a restricted use of these additional tests is justified. Could we rely on DRE and use additional tests only in complex patients?

Another reason to perform anorectal function tests is an attempt to objectively measure the anal pressures. Since there is no gold standard, a reappraisal for DRE by experienced investigators seems worthwhile investigating.

The Proctos Clinic is a tertiary referral center for specialized proctological care with experienced surgeons, a pelvic floor physical therapist, and a fully equipped anorectal function laboratory. The aim of our study was to examine the correlation of the anal pressures between DRE, 3D-HRAM, and the s-EMG. DRE, 3D-HRAM, s-EMG, BET, and the transperineal ultrasound were compared to diagnose dyssynergia. Furthermore, we sought to assess the level of agreement between DRE performed by the surgeon and the pelvic floor physical therapist.

## Methods

### Study population

The Proctos Clinic is a tertiary referral center for anorectal function complaints. Between January 2020 and April 2022, men and women aged 18 to 80 years, who underwent anorectal function tests and were referred for pelvic floor physical therapy (PFPT), were invited to participate in the study. Exclusion criteria were noncompliance with verbal instruction in Dutch and current psychiatric disorders. Patients in whom the timeframe was more than 4 weeks between the tests were excluded as the measurements may not be comparable.

Patients first visited the surgeon, who performed a DRE and a transperineal ultrasound and counseled the patients for the study. Subsequently, patients were asked to participate in case they were referred for 3D-HRAM, BET, and pelvic floor physical therapy. The pelvic floor physical therapist also performed DRE and s-EMG at first visit. The pelvic floor physical therapist was blinded for the DRE of the surgeon and also for the results of the 3D-HRAM, BET, and transperineal ultrasound. All appointments were scheduled within 4 weeks. Results of the different tests were prospectively recorded. All patients signed a written informed consent before entering the study. The study was approved by the Medical Ethics Review Committee of the Amsterdam University Medical Centers, location AMC.

### Anorectal investigations

#### Digital rectal examination

DRE was performed by all five surgeons and the pelvic floor physical therapist in the same standardized way. The procedure of DRE was explained to the patient. During the assessment, the patient was lying on his/her left side with the knees flexed at 90°. The examiners used nonallergic gloves lubricated with water-based gel. All patients were asked to empty their bladder before the assessment. After careful insertion of the index finger, the sphincter tone was assessed at rest and scored as low, normal, or high (Table 1). Squeeze tone was evaluated as the increment in pressure and scored similar. Then, the patient was asked to squeeze for 30 s. The squeeze pressure was scored as low, normal, or high. Subsequently, the examiner placed his/her left hand on the patient’s abdomen and the patient was asked to push and bear down. Push effort was scored as relaxation, indifferent, or paradoxical contraction.

**Table 1:** Summary of anorectal function tests and their categorized outcomes

	Mean resting pressure	Mean squeeze pressure	Push	Evacuation
DRE surgeon	1. Low 2. Normal 3. High	1. Low 2. Normal 3. High	1. Relaxation 2. Indifferent 3. Paradoxical	–
DRE pelvic floor physical therapist	1. Low 2. Normal 3. High	1. Low 2. Normal 3. High	1. Relaxation 2.Indifferent 3. Paradoxical	–
3D-HRAM	1. Low: 0–49 mmHg 2. Normal: 50–100 mmHg 3. High: > 100 mmHg	1. Low: 0–49 mmHg 2. Normal: 50–200 mmHg 3. High: > 200 mmHg	1. Relaxation 2. Indifferent 3. Paradoxical	–
s-EMG	Women 1. Low: 0–2.0 2. Normal: 2.1–5.0 3. High: > 5.1 Men 1. Low: 0–3.0 2. Normal 3.1–6.0 3. High: > 6.1	Women 1. Low: 0–6.0 2. Normal: 6.1–15.0 3. High: > 15.1 Men 1. Low: 0–9.0 2. Normal: 9.1–18.0 3.3.3.3.3.High: > 18.1	1. Decrease of electrical activity (relaxation) 2. Indifferent 3. Increase of electrical activity (paradoxical)	–
Transperineal echo	–	–	1. Relaxation 2. Indifferent 3. Paradoxical	1. Yes 2. No
BET	–	–	–	1. < 1 min = normal 2. > 1 min = abnormal

*DRE* digital rectal examination, *3D-HRAM* 3-dimensional high-resolution anorectal manometry, *s-EMG* surface electromyography, *BET* balloon expulsion test

#### Surface electromyography (s-EMG)

Pelvic floor muscle tone and function were measured with s-EMG (μV) [10] with an intra-anal probe (Maple^®^, Novuqare Pelvic Health B.V. CE 0344, Rosmalen, the Netherlands). This is a probe with a matrix of 24 electrodes enabling measuring s-EMG signals from the different sides and layers of the pelvic floor muscles. The s-EMG probe is placed intra-anal, with the reference electrode placed on the spina iliaca anterior superior. Patients were asked to perform four consecutive tasks: (1) one minute rest where patients were instructed to feel the pelvic floor in rest, (2) three maximum voluntary contractions where patients were instructed to perform a controlled contraction and relaxation of the pelvic floor muscles, (3) one endurance contraction where patients were instructed to contract the pelvic floor muscles at such a level that they could hold for 30 s, and (4) one push effort where the patient was asked to bear down. The examiner was holding the probe to keep it in place. From these s-EMG measurements, mean EMG amplitudes per electrode were calculated. A sustained increase in surface s-EMG activity (> 50% increase from baseline) on attempted bearing down was defined as dyssynergia. The s-EMG values are presented as absolute values (mV). Normal values have not been published yet. For this reason, the pelvic floor physical therapist estimated the normal values for men and women on clinical experience and a recent study where s-EMG values where measured during PFPT in patients with a chronic anal fissure [21] (Table 1). Results of the one-year follow-up will be published shortly.

#### 3D high-resolution anal manometry (3D-HRAM)

**Fig. 1:**
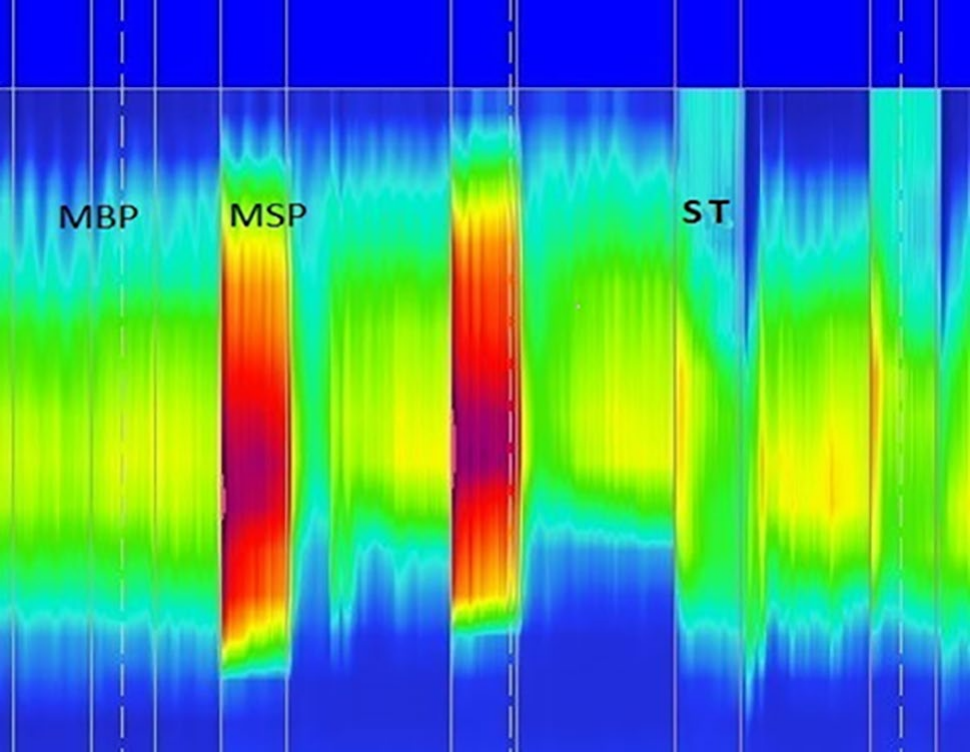
3D-HRAM. Normal pressure profile during rest (MBP), increase during squeeze (MSP), and decrease during straining (ST)

**Fig. 2:**
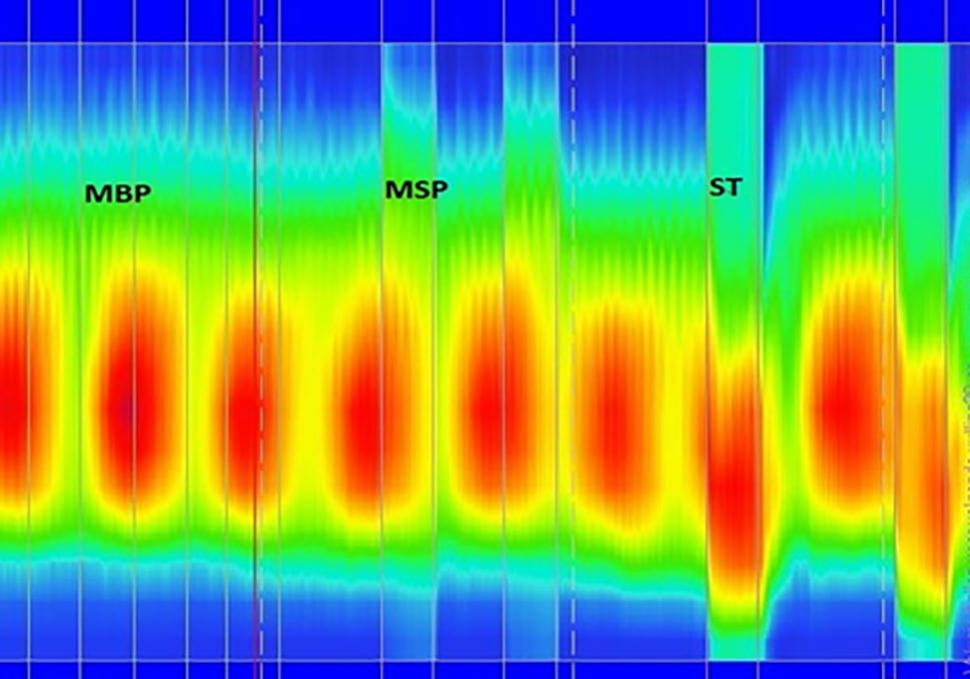
3D-HDRAM. Dyssynergia. A high basal pressure (MBP) profile is seen with no changes in pressure during maximal squeeze (MSP) and straining (ST)

The 3D-HRAM was performed by a nurse continence specialist, and the methods are previously described [22]. The anorectal probe has 256 pressure sensors on 16 lines, each line having 16 circumferential sensors. The probe, which is covered by a disposable sheath, has a diameter of 10.75 mm, a length of 64 mm, and an internal lumen to inflate the balloon (3.3 cm long with a capacity of 400 cc). Patients underwent the test in the left lateral position. Patients were asked to use a MICROLAX^®^ enema the night before and the morning of the test. Pressures were measured at rest, during squeeze, and during straining according to the London protocol (Carrington IAPWG 2019). Analysis of the manometry data was performed with ManoView (Given Imaging, Duluth, GA, USA). The mean resting pressure (MRP) and mean squeeze pressure (MSP) were measured by the software and were additional visually reviewed by the gastroenterologist RF. Figures 1 and 2 show examples of the pressure profile during rest (MBP) and during squeeze (MSP) with ManoView. Normal values have been published by several authors and show a large range [14, 23–28]. Based on these studies, we considered an anal rest or squeeze pressure lower than 50 mmHg as “low.” For comparison with the other tests, the anal pressures were categorized as described in Table 1.

#### The balloon expulsion (BET)

A nonsterile disposable balloon (BARD, Covington, USA) was filled with 50 cc water or until the patients felt a desire to defecate. Balloon expulsion time differs in literature. According to several studies, evacuation within 1 min was considered as normal [28–30]. The BET was performed by a nurse continence specialist in our clinic, and results were scored < 1 min or > 1 min [1] (Table 1).

#### Transperineal ultrasound (TPUS)

This was performed with a standard BK Medical scanner (BK Medical ApS, Herlev, Denmark) and a transducer (BK Medical, type 2C9, 13 MHz). The patient was lying supine with the legs flexed. As with the 3D-HRAM, patients were asked to use a MICROLAX^®^ enema the night before and the morning of their appointment. Transperineal ultrasound was performed using a conventional curved array probe rested on the perineum to gain dynamic two-dimensional midplane sagittal views. For the real-time movement, 50 ml echo lucent gel was introduced in the rectum. The patient was asked to squeeze, bear down, and cough while views were digitally recorded. The movements during straining were categorized as relaxation, indifferent, and paradoxical contraction. Evacuation of gel during straining was categorized as yes or no (Table 1).

### Statistical analysis

All statistical analyses were performed using SPSS (IBM, SPSS Statistics 28). Continuous data were described as mean or median depending on the distribution, including range and standard deviation. Statistical analysis was performed by comparing categorical results of anal pressures with descriptive statistics using crosstabs, namely, the resting and squeeze pressures and straining movement of DRE by the surgeon and pelvic floor physical therapist, 3D-HRAM, s-EMG, transperineal ultrasound (with echo lucent gel), and BET. The interrater agreement for DRE, which included tone during rest and squeeze and straining movement, between the referring surgeon and the pelvic floor physical therapist was assessed by using Cohen’s Weighted Kappa test. Agreement was classified as follows: poor agreement (0.00–0.20), fair (0.21–0.40), moderate (0.41–0.60), substantial (0.61–0.80), and almost perfect agreement (0.81–1.00). *p *values of < 0.05 were considered significant.

## Results

### Patients, demographics, and clinical characteristics

Between January 2020 and April 2022, 56 patients were referred for PFPT by the surgeon and underwent anorectal function tests in the diagnostic workup. Six patients were excluded due to incomplete data because the patient cancelled an appointment or when treatment started between the different tests. The appointment for the 3D-HRAM was always prior to or at the same day as the PFPT.

Demographics and clinical characteristics of the study group are detailed in Table 2. A total of 37 (74%) females were included, and median age was 51 years. Twenty-three (62%) females had two or more vaginal deliveries. Thirty-one (62%) patients previously received PFPT. The most frequent indication for referral for PFPT was fecal incontinence in 27 patients (54%).

**Table 2:** Patient characteristics

	No. patients
Gender	
Male, *n *(%)	13 (27)
Female, *n *(%)	37 (74)
Median age, years (SD)	51 (15)
Indication, *n *(%)	
Fecal incontinence	27 (54)
Obstructed defecation	10 (21)
Chronic anal fissure	3 (6)
Hemorrhoidal disease	2 (4)
Other	8 (17)
Vaginal parity, *n *(%)	
0	7 (19)
1	7 (19)
2	14 (38)
≥3	9 (24)
Rectal surgery in the past, *n* (%)	9 (18)
Radiotherapy in the past, *n *(%)	1 (2)
Urologic or gynecologic surgery in the past, *n*(%)	10 (20)
Neurological or connective tissue disease, *n *(%)	3 (6)
Pelvic floor physical therapy in the past, *n *(%)	31 (62)

### Interrater agreement digital rectal examination

The assessed sphincter tone and pelvic floor muscle function with DRE by the surgeon and the pelvic floor physical therapist during rest, squeeze, and straining correlated in 78%, 78%, and 84%, respectively. This resulted in substantial agreement for assessing the resting tone with Cohen’s Weighted Kappa (κ) of 0.749 (95% CI 0.612–0.886). In the assessment of the squeeze tone, this was somewhat lower, but still substantial, with a (κ) of 0.620 (95% CI 0.432–0.807). When assessing straining, they agreed almost perfect with a (κ) of 0.819 (95% CI 0.700–0.938).

The prolonged squeeze (30 s) was only performed by few surgeons, and therefore, we omitted this variable from the analysis.

### Digital rectal examination by the surgeon and pelvic floor physical therapist and anorectal manometry (*n* = 46 and *n* = 45)

When classifying the resting tone and pressure as low, normal, or high, 23 (47%) patients were assessed similar by the surgeon’s DRE and the 3D-HRAM. In the assessment of squeeze tone and pressures, this was somewhat better with 31 (65%) patients. DRE of the pelvic floor physical therapist was similar to 3D-HRAM in 26 (53%) and 32 (65%) patients in the assessment of the resting and squeeze tone and pressure, respectively.

### Digital rectal examination by the surgeon and pelvic floor physical therapist and surface electromyography (*n* = 49 and *n* = 50)

The resting tone assessed by the surgeon’s DRE and s-EMG activity was similar in only 18 (36%) patients. For squeeze, this was 32 (65%). DRE by the pelvic floor physical therapist correlated in 18 (36%) and 41 (82%) patients with s-EMG in the assessment of resting tone and squeeze tone. The surgeon and the pelvic floor physical therapist both classified the resting tone with DRE in, respectively, three and four patients as “low” while s-EMG activity assessed “high.” One patient with a chronic anal fissure was classified “high” for squeeze tone with DRE by both the surgeon and pelvic floor physical therapist but classified “low” with s-EMG.

### Anorectal manometry and surface electromyography (*n* = 49)

When the results are categorized as low, normal, and high, the 3D-HRAM and s-EMG correlated well in only 18 (37%) patients when comparing the resting pressure and electric activity. With 29 (59%) patients, this was better when comparing the squeeze tone and electric activity. Overall, four patients who were classified as “low” on the 3D-HRAM were classified “high” with s-EMG activity concerning resting pressure and one patient vice versa during squeeze pressure.

### Comparing detecting dyssynergia

- **BET and evacuation of gel during TPUS (*n* = 19)**
  Four patients were not able to evacuate the gel despite being able to expel the balloon within one minute. Three patients evacuated the gel—of whom two not completely—while they were not able to expel the balloon within one minute (Table 3).
- **TPUS and evacuation of gel during TPUS (*n *= 24)**
  Half of the patients who underwent TPUS with echo lucent gel evacuated the gel (Table 4). Nineteen patients were classified as “indifferent” regarding the straining movement.
- **TPUS and BET (*n *= 23)**
  Eighteen patients were classified “indifferent” on the transperineal ultrasound (Table 5). Almost half of them expelled the balloon within one minute and the other half in more than one minute or not at all. One patient showed normal “relaxation” of the puborectalis muscle when straining on TPUS, whereas he was not able to expel the balloon within one minute.
- **s-EMG versus BET (*n *= 37)**
  Thirteen patients (35%) were classified as “paradoxical” of whom almost half was able to expel the balloon within one minute and half could not (Table 6). Fourteen patients were classified as “indifferent” of whom nine was not able to expel the balloon within one minute.
- **3D-HRAM with BET (*n *= 37)**
  Four out of 10 patients (40%) who showed paradoxical straining on the 3D-HRAM were able to expel the balloon within one minute while five out of the 16 patients (31%) who showed normal relaxation could not expel the balloon within one minute (Table 7).
- **DRE by the surgeon versus BET (*n *= 37)**
  Half of the 10 patients who were classified as “indifferent” were able to expel the balloon within one minute (Table 8). Of the patients who were assessed as normal “relaxation” or “paradoxical,” respectively, 9 of 15 (67%) and 4 of 12 (33%) were able to expel the balloon within one minute.
- **DRE by the pelvic floor physical therapist versus BET (*n *= 37)**
  Results are almost similar with the DRE by the surgeon.
- **s-EMG versus TPUS (*n *= 32)**
  Twelve patients (37%) showed the same results concerning classifying the puborectalis muscle movement in these tests (Table 9).
- **s-EMG versus evacuation of gel during TPUS (*n *= 24)**
  Two patients were not able to evacuate the gel while they showed a decrease in electric activity which corresponds with relaxation of the pelvic floor muscles (Table 10). One patient evacuated the gel completely during TPUS but showed an increase in electric activity with the s-EMG. This patient did not show paradoxical movement on the other tests.
- **3D-HRAM versus TPUS (*n *= 32)**
  In 8 (25%) patients, the test showed the same results (Table 11). TPUS was often classified as “indifferent” in 25 (78%) patients.
- **3D-HRAM versus evacuation of gel during TPUS (*n *= 24)**
  Two patients were classified as “paradoxical” but were able to evacuate the gel during TPUS (Table 12). Also, three patients could not evacuate while they showed normal “relaxation” on the 3D-HRAM.
- **3D-HRAM versus s-EMG (*n *= 50)**
  Twenty-six (52%) patients showed similar results in both tests (Table 13). s-EMG was more often classified as “indifferent” and one patient was classified “paradoxical” while normal “relaxation” was measured using 3D-HRAM.
- **TPUS versus DRE by the surgeon (*n *= 32)**
  In 17 patients (52%), the tests showed similar results. Twenty-five (78%) patients were classified “indifferent” with TPUS (Table 14).
- **TPUS versus DRE by the pelvic floor physical therapist (*n *= 32)**
  Results are almost similar with the DRE by the surgeon.
- **DRE by the surgeon versus evacuation of gel during TPUS (*n *= 24)**
  One patient showed “paradoxical” straining during DRE by the surgeon but could evacuate the gel during the TPUS at the same day (Table 15). One patient could not evacuate the gel while the surgeon classified “relaxation” with DRE.
- **DRE by the pelvic floor physical therapist versus evacuation of gel during TPUS (*n *= 24)**
  Results are almost similar with DRE by the surgeon except that DRE in two patients were classified as “relaxation” while they could not evacuate the gel.
- **s-EMG versus DRE by the surgeon (*n *= 50)**
  In 26 (52%) patients, the test results were similar. s-EMG classified “indifferent” in 22 (44%) patients (Table 16). One patient was classified “paradoxical” with s-EMG but classified “relaxation” by the surgeons’ DRE.
- **s-EMG versus DRE by the pelvic floor physical therapist (*n *= 50)**
  In 31 (62%) patients, the test results were similar (Table 17).
- **3D-HRAM versus DRE by the surgeon (*n *= 50)**
  In 26 (52%) patients, the test results were similar (Table 18). Five patients were classified as “paradoxical” straining by the surgeon while these patients showed “relaxation” on 3D-HRAM. The other way around, one patient was classified “paradoxical” with 3D-HRAM but the surgeon classified DRE as “relaxation.”
- **3D-HRAM versus DRE by the pelvic floor physical therapist (*n *= 50)**
  Results were almost similar to the surgeon’s DRE.

**Table 3:** Balloon expulsion test (BET) versus evacuation of gel during transperineal ultrasound (TPUS)

		Evacuation of gel during TPUS		
		Yes	No	Total
BET	< 1 min	5	4	9
	> 1 min	3	7	10
Total		8	11	19

**Table 4:** Transperineal ultrasound (TPUS) versus evacuation of gel during TPUS

		Evacuation of gel during TPUS		
		Yes	No	Total
TPUS	Relaxation	3	0	3
	Indifferent	9	10	19
	Paradoxical	0	2	2
Total		12	12	24

**Table 5:** Transperineal ultrasound (TPUS) versus balloon expulsion test (BET)

		BET		
		< 1 min	> 1 min	Total
TPUS	Relaxation	1	3	4
	Indifferent	10	8	18
	Paradoxical	0	1	1
Total		11	12	23

**Table 6:** Surface electromyography (s-EMG) versus balloon expulsion test (BET)

		BET		
		< 1 min	> 1 min	Total
s-EMG	Relaxation	7	3	10
	Indifferent	5	9	14
	Paradoxical	6	7	13
Total		18	19	37

**Table 7:** 3D high-resolution anorectal manometry (3D-HRAM) versus balloon expulsion test (BET)

		BET		
		< 1 min	> 1 min	Total
3D-HRAM	Relaxation	11	5	16
	Indifferent	3	8	11
	Paradoxical	4	6	10
Total		18	19	37

**Table 8:** Digital rectal examination (DRE) of the surgeon versus balloon expulsion test (BET)

		BET		
		< 1 min	> 1 min	Total
DRE surgeon	Relaxation	9	6	15
	Indifferent	5	5	10
	Paradoxical	4	8	12
Total		18	19	37

**Table 9:** Surface electromyography (s-EMG) versus transperineal ultrasound (TPUS)

		TPUS			
		Relaxation	Indifferent	Paradoxical	Total
s-EMG	Relaxation	2	6	0	8
	Indifferent	3	9	1	13
	Paradoxical	0	10	1	11
Total		5	25	2	32

**Table 10:** Surface electromyography (s-EMG) versus evacuation of gel during transperineal ultrasound (TPUS)

		Evacuation of gel during TPUS		
		Yes	No	Total
s-EMG	Relaxation	6	2	8
	Indifferent	5	4	9
	Paradoxical	1	6	7
Total		12	12	24

**Table 11:** 3D high-resolution anorectal manometry (3D-HRAM) versus transperineal ultrasound (TPUS)

		TPUS			
		Relaxation	Indifferent	Paradoxical	Total
3D-HRAM	Relaxation	3	13	0	16
	Indifferent	2	3	0	5
	Paradoxical	0	9	2	11
Total		5	25	2	32

**Table 12:** 3D high-resolution anorectal manometry (3D-HRAM) versus evacuation of gel during transperineal ultrasound (TPUS)

		Evacuation of gel during TPUS		
		Yes	No	Total
3D-HRAM	Relaxation	8	3	11
	Indifferent	2	3	5
	Paradoxical	2	6	8
Total		12	12	24

**Table 13:** 3D high-resolution anorectal manometry (3D-HRAM) versus surface electromyography (s-EMG)

		s-EMG			
		Relaxation	Indifferent	Paradoxical	Total
3D-HRAM	Relaxation	9	13	1	23
	Indifferent	3	5	3	11
	Paradoxical	0	4	12	16
Total		12	22	16	50

**Table 14:** Transperineal ultrasound (TPUS) versus digital rectal examination (DRE) by the surgeon

		DRE surgeon			
		Relaxation	Indifferent	Paradoxical	Total
TPUS	Relaxation	5	0	0	5
	Indifferent	6	10	9	25
	Paradoxical	0	0	2	2
Total		11	10	11	32

**Table 15:** Digital rectal examination (DRE) by the surgeon versus evacuation of gel during transperineal ultrasound (TPUS)

		Evacuation of gel during TPUS		
		Yes	No	Total
DRE surgeon	Relaxation	7	1	8
	Indifferent	4	5	9
	Paradoxical	1	6	7
Total		12	12	24

**Table 16:** Surface electromyography (s-EMG) versus digital rectal examination (DRE) by the surgeon

		DRE surgeon			
		Relaxation	Indifferent	Paradoxical	Total
s-EMG	Relaxation	9	3	0	12
	Indifferent	7	8	7	22
	Paradoxical	1	6	9	16
Total		17	17	16	50

**Table 17:** Surface electromyography (s-EMG) versus digital rectal examination (DRE) by the pelvic floor physical therapist

		DRE pelvic floor physical therapist			
		Relaxation	Indifferent	Paradoxical	Total
s-EMG	Relaxation	12	0	0	12
	Indifferent	6	10	6	22
	Paradoxical	0	7	9	16
Total		18	17	15	50

**Table 18:** 3D high-resolution anorectal manometry (3D-HRAM) versus digital rectal examination (DRE) by the surgeon

		DRE surgeon			
		Relaxation	Indifferent	Paradoxical	Total
3D-HRAM	Relaxation	12	6	5	23
	Indifferent	4	5	2	11
	Paradoxical	1	6	9	16
Total		17	17	16	50

## Discussion

The present study provides an overview of the correlation between outcomes of frequently performed anorectal function tests and compares their ability to measure dyssynergia. Furthermore, this study measured the level of agreement between DRE performed by the surgeon and the pelvic floor physical therapist in a tertiary referral center.

Despite the surgeons and the pelvic floor physical therapist being experienced, performing several digital rectal examinations per day, the agreement of the anal tone between their DRE was not perfect. The assessed tone during rest, squeeze, and straining did not correlate in 22%, 22%, and 16%, respectively. To the best of our knowledge, no literature concerning the interrater agreement of DRE has been published. Interrater agreement has only been studied in vaginal digital assessment concerning the pelvic floor function and digital rectal examination in the context of prostate cancer [31–33]. Overall, the agreement was substantial to almost perfect. The small differences in classification of DRE between the surgeon and pelvic floor physical therapist may be explained by differences in interpretation of the indifferent movement of the pelvic floor. Not a single examination was classified both as relaxation and paradoxical movement.

The correlation between the surgeons’ DRE, pelvic floor physical therapists’ DRE, and the 3D-HRAM in our study was moderate and somewhat better for squeeze tone/pressures than resting tone/pressures. Several studies compared DRE with ARM and showed an overall good agreement of pressures, however similar to our study, slightly better for squeeze pressures, but results are not consistent [9, 15, 34–39]. For example, the study by Beatrice et al. showed that DRE correlates well, but not perfectly, with the ARM for resting pressures, (*r *= 0.71 (*p* < 0.001) [9]. However, Orkin et al. observed an excellent agreement between DRE and the ARM for resting pressures (*r *= 0.82) and for squeeze pressures (*r *= 0.81) [34]. In contrast, Soh et al. described a poor agreement between DRE and ARM for resting pressures with a k coefficient of 0.01 and a moderate agreement for squeeze pressure with a k coefficient of 0.42 [35]. Pinto et al. showed a moderate to strong agreement for resting pressure with a Gamma index of 0.7 and a strong correlation of the squeeze pressures with a Gamma of 0.96 [37]. All studies—including ours—report that the examinations were performed by experienced examiners but the results vary considerably. Nevertheless, ARM can be performed with a variety of types of equipment, techniques, and study protocols, making results less reproducible and thus difficult to compare [40, 41]. A recent study by Prichard et al. described even significantly different results during ARM between operators despite using similar instructions to patients [16]. Even a small difference in outcome could lead to a different interpretation. It must be noted that in contrast to most ARM studies we used the 3D probe.

DRE correlated better with 3D-HRAM in patients referred for fecal incontinence. With 54%, this was the largest group in this study. However, defining “normal” resting and squeeze pressures for ARM values is quite difficult. There is obviously an overlap since several studies showed different values for normal and abnormal resting and squeeze pressures for ARM [14, 23–28]. To be accurate in comparing between groups, the pressures should be adjusted according to age, gender, and parous and nulliparous females. But these differences were small, and to make comparisons between tests manageable in this study, we did not differentiate.

The surgeons’ DRE and the pelvic floor physical therapist’s DRE were compared to the s-EMG and showed some discrepancies. The surgeon’s DRE and the pelvic floor physical therapist’s DRE were categorized as “low” whereas the s-EMG categorized “high” in three and four patients, respectively. However, one patient was categorized “high” with DRE and “low” with s-EMG. This can probably be explained by the fact that patients who can hardly control their external anal sphincter might overcompensate with their levator muscle. As we measured with s-EMG, the mean of the total electrical activity of the external anal sphincter including the levator muscle, the EMG activity might be higher than expected. When retrospectively assessing the 3D-HRAM, these patients showed indeed higher pressures of the posterior levator muscle on the 3D image in contrast to the sphincter and vice versa for the patient with a chronic anal fissure. Furthermore, high tone on the levator muscle with DRE might be turgor which is not measured with s-EMG. For this reason, comparing s-EMG with other tests might not be appropriate and should probably be used only to confirm physical examination and biofeedback registration.

The correlation between s-EMG and the 3D-HRAM was better for squeeze pressures and electric activity than resting pressures and electric activity with an agreement of 59% and 37%, respectively. A study from 1989 also showed limited concordance with a correlation coefficient of 0.55 (*p* < 0.001) between the maximum squeeze pressure with ARM and maximum contraction pattern with the EMG [17]. Regarding diagnosing dyssynergia while straining with s-EMG and 3D-HRAM, our results were not in line with the results by Chiarioni et al. [30]. In our study, s-EMG and ARM were concordant in 52% while Chiarioni et al. described an agreement of 88% for classifying patients’ dyssynergic or not dyssynergic. Both tests are used to test the anorectal function but are used for different purposes in clinical practice. The question that remains is how relevant small differences are in clinical practice.

The results of the six different function tests used to diagnose pelvic floor dyssynergia, namely, DRE by both the surgeon and the pelvic floor physical therapist, 3D-HRAM, s-EMG, BET, and transperineal ultrasound (with echo lucent gel), were to some extent comparable. Although most comparisons were statistically significant, the correlation remained low. Discrepancies with TPUS could be explained by the nonanatomical supine position of the test and the fact that the patient is not in private environment. Three patients who evacuated the gel—although not completely—but were not able to expel the balloon within 1 min were referred for PFPT because of fecal incontinence. It is very likely that these patients lost the gel by leaking, not because of the push effort. This makes these tests not suitable to compare.

Furthermore, the tests are performed in different postures; the balloon expulsion is performed in a private setting, in sitting position, whereas the other tests are performed by an examiner with the patient lying in the left lateral position. 3D-HRAM measures the anorectal pressures, s-EMG measures electrical activity, and TPUS is visually assessed by the doctor where evacuating echo lucent gel might support their findings. Some discrepancies cannot be explained except the snapshot nature of the tests. It is known that the diagnostic accuracy of ARM is limited for discriminating between healthy people and patients with functional constipation [42]. Unfortunately, previous studies with TPUS assessed its accuracy for detecting rectocele, intussusception, or enterocele or used a total pelvic floor ultrasound without echo lucent gel. No previous studies reported its accuracy to diagnose dyssynergia. However, based on our experience, the TPUS is a low-cost and easy tool for surgeons to perform. Surgeons are able to perform their own test in the outpatient clinic, and moreover, it has comparable results with the classical defecography [43] which makes it worth considering a relevant anorectal function test. The BET is a frequently used test for assessing defecatory dysfunction since it is a simple and low-cost procedure. Different protocols are used to perform the procedure, air filled or water-filled balloon, lying or seated position. Time values that are considered abnormal range from 1 till 5 min [28–30, 44, 45]. In our study, a balloon expulsion time of more than 1 min was considered prolonged. This was categorized as dyssynergia by the 3D-HRAM in 32% of the cases. In contrast to older studies, more recent studies demonstrated poor agreement between BET and ARM [46, 47].

According to the ROME IV criteria, dyssynergic defecation is established by two out of three tests: (1) ARM or s-EMG, (2) balloon expulsion test, or (3) defecography. Remarkable is that the ARM or the s-EMG should be abnormal and that DRE and the transperineal ultrasound are not mentioned in this workup [7]. This might be confusing and suggests that none of the tests can be considered as golden standard. Furthermore, anorectal function tests provide additional workload and costs whereas DRE is widely available and dyssynergia is a widespread phenomenon. The ROME IV criteria are merely used to standardize patients in an attempt to objectivize dyssynergia. Also, Bordeianou et al. had their doubts about which test to assign the highest value, the s-EMG, BET, or ARM, prior to referral to the pelvic floor physical therapist with dyssynergia [48].

Undoubtedly, this study has a number of limitations which should be acknowledged. First, the surgeons and the pelvic floor physical therapist were unblinded to the patients’ medical history when performing the DRE which likely has influenced the results by information bias. Secondly, although all surgeons and the pelvic floor physical therapist were given instructions before the study started on how to perform a complete structured DRE and systematically describe the physical examination in the electronic health record, variety in performing and assessing DRE is insurmountable. The single observer for all 3D-HRAM results might be a lowness or a strength in this study. A considerable limitation of this study is that we were not able to use controlled normal s-EMG values since they have not yet been published. Furthermore, the results of the study would have had more relevance if there was a gold standard or known sensitivity of the tests. This issue is also reflected in the ROME IV criteria for dyssynergic defecation as mentioned above. Unfortunately, not all patients underwent all tests due to logistic problems in the outpatient clinic concerning the tests in the context of the study. Consequently, some patients did not undergo the BET or the TPUS. Lastly, there might have been interpretation bias by assessing straining movement of the pelvic floor. It is not known how “indifferent” movement of the pelvic floor is defined among the examiners; does this mean “no movement” or also “relaxation but not enough”? This probably resulted in different outcomes.

This study showed that squeeze pressures were more often similarly categorized than resting pressures in anorectal function tests. It further shows that the surgeons’ DRE and the pelvic floor physical therapist’s DRE more often similar assessed in comparison to anorectal functions tests as 3D-HRAM, s-EMG, or TPUS. Still, the correlation between all tests is quite disappointing and this raises questions regarding when to perform these tests in addition to DRE. Or does this mean that we can suffice with an expert’s DRE when referring to the pelvic floor physical therapist for dyssynergia? The pelvic floor physical therapist will evaluate therapy with his/her own DRE with or without s-EMG, not with ARM or transperineal ultrasound. Perhaps we should only perform anorectal function tests in patients who are refractory to conservative treatment like lifestyle and pelvic floor physical therapy or when more invasive procedures like surgery or Botox, e.g., are considered. Furthermore, these tests are valuable when evaluating new (surgical) therapies.

## Conclusion

This study shows that DRE has a good correlation among experienced investigators. Since commonly performed anorectal function tests correlate poorly with DRE and with other anorectal function tests, DRE by an experienced investigator suffices in daily clinical practice. When conservative treatment fails, further investigation is warranted; however, these results should be interpreted with caution.

## Data Availability

Our data is available on reasonable request.
